# Pathways to sustainability: Higher education and green productivity

**DOI:** 10.1371/journal.pone.0318619

**Published:** 2025-02-24

**Authors:** Yongchun Sun

**Affiliations:** School of Economics and Management, Guangzhou Nanyang Polytechnic College, Guangzhou, Guangdong, China; Sejong University, KOREA, DEMOCRATIC PEOPLE'S REPUBLIC OF

## Abstract

The research conducted theoretical analysis and empirical testing on the relationship between higher education and regional green productivity based on panel data from 30 Chinese provinces from 2003 to 2021. The study’s findings demonstrate that higher education can have a major impact on local green production. In order to determine whether industrial structure upgrading and the digital economy work together to promote the development of green productivity, higher education is added to these factors at the same time as the new economic growth mode transformation in the digital economy era. The research hypothesis aligns with the results, suggesting that higher education and the digital economy collaborate to enhance green productivity levels. Higher education has a more significant impact on green productivity the greater the level of regional economic growth, according to a further nonlinear test utilizing the partial linear function coefficient (PLFC) model. Higher education’s influence on green production varies by place and period, becoming more pronounced as time passes and the degree of regional economic growth rises. In order to fully utilize higher education’s capacity for scientific research, innovation, and talent, as well as to increase the direct contribution of its scientific and technological innovations to the advancement of national industry and production promotion, it is imperative that people actively promote the new type of industrialization, develop the digital economy, and work in tandem with higher education.

## 1. Introduction

The notion of green growth was first introduced in the 2005 Asia-Pacific Ministerial Declaration on Environment and Development, which committed to collaboratively attain a mutually beneficial outcome for environmental preservation and economic advancement, thereby achieving sustainable development defined by “green growth.” Green development, a concept associated with green growth, has been frequently referenced in international agreements, including the United Nations Environment Programme, various multilateral development institutions, the United Nations Framework Convention on Climate Change, and the Paris Agreement, all of which underscore the necessity for global cooperation and the collective advancement of green development through multilateral mechanisms. Green development is not the province of any one nation or area; rather, it depends on international collaboration, the exchange of technological advancements and knowledge, and the establishment of an environment that is conducive to collaboration in order to collectively tackle the problems that humanity faces. China actively engages in and significantly contributes to the theory and practice of green development, offering the Chinese plan for green development through the establishment of an ecological civilization. In its report to the Nineteenth National Congress, the Communist Party of China (CPC) suggested implementing the new development concept and establishing a green mode of production and lifestyle. “The 14th Five-Year Plan proposes a new pattern of economic development based on two main lines: increasing total factor productivity and developing a green economy.” The report of the 20th National Congress specifically proposes “promoting green development and harmonious coexistence between humans and nature.” Green development has emerged as the new era’s theme, owing to the government’s emphasis on ecological priorities. How to balance economic development and environmental protection has emerged as a critical factor in reforming China’s economic development mode, and it is also a topic of active research around the world [1]. Traditional total factor productivity measures cannot effectively reflect China’s economic development. At this stage of China’s high-quality development, people should focus more on green productivity, which takes into account both resource inputs and environmental outputs. Green productivity accounts for the input of production factors as well as the consumption of energy resources, alters China’s GDP-oriented economic development model, increases endogenous motivation for energy-saving and emission-reduction behaviors, and serves as an effective and comprehensive indicator of high-quality economic development in the new era.

Human capital is an essential component of promoting green growth. Human capital is the primary driver of economic growth, according to Schultz’s theory of human capital [2]. Higher education (HE), which is the most fundamental and essential component of human capital, can raise its value by improving workers’ knowledge and abilities. It also serves as a major driver of technological advancement, which is critical for improving the effectiveness and quality of economic growth. In addition, human capital plays a significant externally positive role in education. Highly educated scientists and technologists, for instance, encourage the conversion of resources through technological innovation, bringing new materials, processes, and technologies to improve economic efficiency; entrepreneurial human capital, through resource allocation optimization; and government administrative human capital, through resource utilization enhancement and other means, to generate new productivity, boost output efficiency, and encourage economic growth. At the same time, highly educated human capital is more likely to expand environmental protection activities and enforce environmental regulations. These studies reaffirm the “pollution paradise” idea by advocating for green development through energy conservation and efficiency [3,4]. As a result, increasing education spending and improving educational standards are critical for achieving steady, high-quality economic growth. To convert its vast human resources into high-quality human capital, China needs higher education. Furthermore, a number of Chinese academics have discovered that innovation in science and technology has not been as significant a driver of green total factor production as higher education. Therefore, it is extremely important to study the effect, mechanism, and trajectory of higher education on green total factor productivity.

The research’s originality and contribution can be summarized in three points: First, this paper considers resource and environmental indicators, which distinguishes it from other green total factor productivity evaluation indicators. Additionally, the desired output indicators include a new indicator of green coverage that reflects the population’s quality of life, in addition to an increase in pollutant indicators for non-desired outputs. All of these factors combine to make the evaluation of green productivity more thorough and logical. By examining the effects of the digital economy and upgrading industrial structures on the connection between higher education and green total factor productivity, it also contributes to the enrichment of related research. There are some gaps in the research on the association between higher education and green productivity, whereas the majority of current studies focus on the relationship between education and economic growth. Third, earlier research on human capital or higher education ignored the notion of neither absolute linear nor absolute nonlinear correlations between variables. In order to investigate the effect of higher education on green productivity under spatiotemporal heterogeneity, this paper uses the PLFC model, which incorporates both linear and nonlinear interactions. This is a more methodical approach to researching this topic.

The remainder of the study is organized as follows: the research hypotheses and literature review are presented in the second part. Variable selection and empirical technique are covered in the third section. Data sources and descriptive statistics make up the fourth section. The study’s findings are presented in the fifth part. The conclusions and suggestions are covered in the sixth part.

## 2. Literature review and research hypothesis

Given the scarcity of resources and the growing severity of environmental pollution, the concepts of green development and sustainable development have gained traction, and resources and the environment are no longer just endogenous variables influencing economic development [5], but have become rigid constraints limiting the quality of economic development. This has led to an increased focus on the concepts of green development and sustainable development. The green total factor productivity is the result of extensive research by numerous scholars, using environmental pollution and resource consumption as indicators and incorporating them into the total factor productivity measurement system to assess economic growth and industrial development [6–10]. With energy, resources, and the environment considered as binding factors of production, the Green Total Factor Productivity (GTFP) measurement method is a more scientific approach that fosters green productivity, enhances economic management, encourages the modernization and transformation of traditional industries, and fosters the coordinated development of the economy, society, and environment. According to [11], green total factor productivity is a critical indicator of the transformation and advancement of green productivity, as well as a reflection of China’s economy’s high-quality and sustainable development. Thus, the purpose of this study is to investigate strategies and mechanisms of action for increasing green productivity. A review of the literature reveals that, in contrast to previous studies, there are several mechanisms for promoting higher education on green productivity. In the current phase of China’s high-quality development, higher education fosters the country’s economy’s sustainable growth through a variety of channels, including direct, complementary, and indirect effects. Next, the relationship between higher education and green economic development is theoretically examined, and pertinent hypotheses are developed in the following section.

Higher education is a key factor in raising labor productivity, helping to bridge the gap between education and economic growth [12]. defined the “slow surplus” as a rise in labor productivity. In order to support the idea that education directly contributes to economic development, scholar [13] included education as a separate input factor into the economic growth model. He made the assumption that technology follows education. The higher education sector, as a significant component of the service sector, directly contributes to the expansion of the national economy by combining capital and labor contributions, building infrastructure, and attracting education-related demand. The Communist Party of China’s report from its twentieth congress, in particular, makes clear that “strengthening the support of human resources for modernization” is a priority. This indicates that the Chinese government fully understands that developing high-quality human capital is essential to achieving high-quality economic development. Colleges and universities are the primary means of raising the caliber of human capital because they combine science and technology as the primary productive force, talent as the primary resource, and creativity as the primary driving force. While there is a wealth of academic research on the influence of human capital on green productivity, there is relatively little on the direct relationship between higher education and green productivity. For example, researchers [14] contend that variations in human capital accumulation account for the majority of the variances in green development and innovation potential among nations or regions. The research can group the channels of influence into four categories: technological progress spillovers [15]; knowledge spillovers [16]; matching the upgrading of the industrial structure [17]; and boosting environmental awareness [18]. Therefore, human capital is a major driver of technical advancement and is crucial for enhancing the effectiveness and caliber of economic growth. However, through energy conservation and efficient usage, human capital has a significant positive externality that might support green development [3,4]. The key to realizing China’s economic shift from the “demographic dividend” to the “talent dividend” and the engine of human capital accumulation is higher education, which has the greatest impact on human capital. Therefore, studying the relationship between increased education and green productivity is essential. This paper advances the following theories, drawing from the aforementioned debate:

*Hypothesis H1: The higher education sector itself can facilitate the growth of green production.*

An effective correspondence between the structure of higher education and the structure of industry is an implicit prerequisite for higher education to be able to strongly contribute to economic development. Higher education that is compatible with the industrial structure can only serve to strengthen the role of higher education in economic growth. Overall, China’s human capital structure is moving from a low level to a high level in tandem with the process of optimizing and upgrading the country’s industrial structure, and both processes are following a consistent evolutionary pattern. Specifically, the upgrading of the industrial structure is essentially to change the situation in which economic growth is highly dependent on resource consumption and brings about pollution, to fundamentally curb the destructive effect of economic growth on the ecological environment, and to realize the harmonious development of the economy and the environment [19]. argue that upgrading industrial structures can effectively improve eco-efficiency through resource integration and environmental pollution reduction [20]; also note that as pollution emissions increase overall, upgrading industrial structures can maximize support for stable social development and help unleash the potential for green economic growth. That is to say, the group of people who receive higher education has a higher ability to integrate resources and stem the secondary school, bringing advanced and efficient management concepts, which in turn promote industrial upgrading. Similarly, the human capital that corresponds to this is more inclined to increase environmental protection efforts to implement environmental protection standards. High-quality human capital can also hasten the spread and flow of knowledge and technology within the region, resulting in the elimination of some outdated industries, the development of new, high-end industries, and the driving force behind industrial upgrading [21]. However, upgrading the industrial structure also raises the demand for higher education. The “polluted paradise” theory further supports the idea that the caliber of human capital in a community has a direct impact on eco-efficiency [22]. highlights that optimal resource allocation necessitates the pairing of cutting-edge technology with highly skilled personnel to achieve the best possible impact. Therefore, combining and improving higher education with industrial structure upgrading is the only way China’s economy can grow and undergo a greener transformation.

Furthermore, as a result of the ongoing advancements in digital information technology, the digital economy is revolutionizing society’s modes of production and consumption in a number of ways. As a result, it has quickly emerged as a major force behind the promotion of green city development, and the achievement of maximum economic output with the least amount of resource consumption and environmental harm. The foundation of the digital economy is highly qualified human resources, even though education, research, and development are the primary ways to realize it. The concept of the digital economy emerged in the early 1980s, distinguishing it from traditional economic structures that primarily rely on human resource-driven economic growth [23]. Talent is the initial resource for development in the digital economy’s wave of change, and ability is essentially what drives the economy’s innovation. The growth of the digital economy reduces barriers to technology and communication, improves resource accessibility, and speeds up the flow of innovative factors like talent, capital, information, and technology. These factors help bridge the digital divide and form the multiplier effect of data on other innovative factors, which in turn helps the green economy develop [24]. Because human capital is the most dynamic and creative factor in production, it is necessary to support the development of the digital economy, among many other factors. According to [25], the lack of human capital to meet the demands of the digital economy’s development will negatively impact both the pace and performance of the economy’s growth [26]. A company can only truly maximize production and make excellent use of its managerial and technological resources when its human capital has reached a specific level. The relationship between economic activity and environmental pollution is significant. We should be more concerned about enhancing environmental awareness, as this not only promotes the development of the green economy but also directs the establishment of related disciplines in colleges and universities and the continued cultivation of talent. Simultaneously, the enhancement of human capital quality has led to an improvement in people’s environmental awareness. Consequently, the digital economy moderates the contribution of higher education to green productivity [27]. The following hypothesis is put forth in this study based on the debate above.

*Hypothesis H2a: The complementarity between industry structural upgrading and higher education can aid in the development of green productivity.*
*Hypothesis H2b: Synergies between higher education and the digital economy may enhance green productivity.*

Regional disparities in economic development persist in China, despite the country’s economy as a whole continuing to expand. For example, in 2015, the northeastern provinces had a per capita GDP of almost 65% of the eastern provinces, whereas the central and western provinces had per capita GDPs of only roughly 52% and 50% of the eastern provinces, respectively. The quality of human capital varies greatly between regions due to interregional disparities in health care, education quality, and other factors caused by uneven economic development. Low-economic- development regions, in particular, have limited resources, yet require substantial resources for green technological innovation. Consequently, doing creative R&D is more difficult in regions with lower economic development levels [28]. This has an impact on the upgrading of regional economies and the green transformation of those regions. However, some researchers have discovered that there is a considerable degree of regional variation in the influence of human capital on the increase in total factor productivity across various locations. For instance [29], found, based on the framework of endogenous growth theory, that we cannot generalize the impact of human capital accumulation on total factor productivity (TFP) and must determine it in the context of the actual situation [30]. For instance, human capital accumulation has a negative effect on TFP growth in developed countries; however, it has a positive effect in middle-class and developing countries, and it positively impacts TFP growth in poor countries that are open to trade. It also benefits underdeveloped nations that allow trade to flourish. This study proposes the following theories based on the previous discussion:

*Hypothesis H3: The impact of higher education on green productivity is non-linear, diverse, and spatiotemporal.*

## 3. Research methodology and variable selection

### 3.1. Dependent variable: Green productivity

[11] state that ecological pressures and resource limitations constrain green productivity, leading to the selection of green total factor productivity (GTFP) as a measure. The test taker must investigate the achievement of sustainable economic growth from both TFP’s and GTFP’s perspectives. Naturally, advancements in measurement methodology, which factor in undesired output while determining total factor productivity, make this kind of decision easier. Human capital, research, and development are also inputs that go into creating GTFP. However, the creation of GTFP also incorporates negative outputs such as the industrial “three wastes” and the green coverage rate of each city, which serves as a gauge of the citizens’ quality of life. The indicators for measuring green productivity are shown in the Table 1.

**Table 1:** Variables for estimating GTFP.

Factors	Variables	Descriptions
Inputs	Capital	Capital stock (in 100 million 2000 CNY)
	Labor	Employment (in 10 thousand persons)
	Energy	Energy consumption (*10000 tons of SCE*)
Desirable output	GDP	Real gross domestic product (in 100 million 2000 CNY)
Undesirable output	Waste water	Total industrial waste water (t*en thousand tons*)
	Solid waster	Industrial solid waste (t*en thousand tons*)
	SO_2_	Industry SO_2_ emissions *(t*en thousand tons)
	Green Ratio	Green coverage rate (percentage)

To calculate the green total factor productivity level, the Non-Radial Non-Angular Data Envelopment Analysis Super Slack Based Measure (DEA-SBM) model [31], which incorporates non-desired output, and the Global Malmquist- Luenberger (GML) [32] index model are used in this work. The Data Envelopment Analysis (DEA) approach, a non-parametric productivity index method developed by [33] in the 1970s, is very innovative in its capacity to evaluate multi-output, multi-input manufacturing operations. DEA has now become one of the most common methodologies for measuring green total factor productivity. This method went through two stages: the directional distance function from the output perspective [34] and the non-radial, non-angle Slack Based Measure (SBM) model [35]. Furthermore, the non-radial, non-angle SBM model efficiently overcomes the inadequacies of the output angle’s directional distance function, as well as properly accounting for slack in the input-output variables. The subsequently developed super-efficient SBM model has an advantage over the SBM model in that it allows for additional comparisons of decision units with an efficiency value of one; however, the measured efficiency is static, and the efficiency values of the decision units are not comparable in the time series. As a result, dynamic measures were introduced, and the Malmquist index, Malmquist-Luenberger (ML) index [36], and Global Malmquist- Luenberger (GML) index [37] developed, respectively. Furthermore, the ML index addresses the Malmquist index’s inability to deal symmetrically with desired and undesired outputs, whereas the GML index solves the problem of linear programming misinterpretation, which is difficult to overcome using the ML index. With reference to [38]’s methodology, first, the production likelihood set is determined. Let DMU_k_ be the decision unit for each Chinese province, where k is the number of Chinese provinces and x = (x_1_,…,x_n_) is the number of factors of production invested by each province, where x ∈ $R_{N}^{+}$ The production process will produce M types of desired outputs, y = (y_1_,…,y_n_) ∈ $R_{M}^{+}$ and I types of undesired outputs, b = (b_1_,…,b_n_) ∈ $R_{I}^{+}$ and represent the inputs and outputs in period t by (x^kt^, y^kt^, b^kt^) [39]. If P^t^(x) represents the set of production possibilities for the current time, then:


$$P^{t}\left(x\right)=\left\{\begin{array}{c} \left(y^{t},b^{t}\right):\overset{K}{\underset{k=1}{\sum }}z_{k}^{t}y_{km}^{t}\geq y_{km}^{t},\forall m;\overset{K}{\underset{k=1}{\sum }}z_{k}^{t}b_{km}^{t}=b_{ki}^{t},\forall i;\\ \overset{K}{\underset{k=1}{\sum }}z_{k}^{t}x_{kn}^{t}\leq x_{kn}^{t},\forall n;\overset{K}{\underset{k=1}{\sum }}z_{k}^{t}=1,z_{k}^{t}\geq 0,\forall k \end{array}\right\}$$ (1)

The weight of cross-sectional data in Eq. 1 is represented by $z_{k}^{t}$ If $z_{k}^{t}\geq 0$ indicates a constant return to scale, then $\overset{K}{\underset{k=1}{\sum }}z_{k}^{t}=1,z_{k}^{t}\geq 0$ indicates a variable return to scale [40]. The $P_{\left(x\right)}^{t}$ model is vulnerable to regression in production technology, though. Some researchers have created the domain-wide production possibility set $P_{\left(x\right)}^{G}$ based on $P_{\left(x\right)}^{t}$, which stresses the comparability and consistency of production frontiers, in order to prevent this violation of reality [37,39]. The model of $P_{\left(x\right)}^{G}$ is as follows:


$$P_{\left(x\right)}^{G}=\left\{\begin{array}{c} \left(y^{t},b^{t}\right):\overset{T}{\underset{t=1}{\sum }}\overset{K}{\underset{k=1}{\sum }}z_{k}^{t}y_{km}^{t}\geq y_{km}^{t},\forall m;\overset{T}{\underset{t=1}{\sum }}\overset{K}{\underset{k=1}{\sum }}z_{k}^{t}b_{ki}^{t}=b_{ki}^{t},\forall i;\\ \overset{T}{\underset{t=1}{\sum }}\overset{K}{\underset{k=1}{\sum }}z_{k}^{t}x_{kn}^{t}\leq x_{kn}^{t},\forall n;\overset{T}{\underset{t=1}{\sum }}\overset{K}{\underset{k=1}{\sum }}z_{k}^{t}=1,z_{k}^{t}\geq 0,\forall k \end{array}\right\}$$ (2)

The Malmquist-Luenberger (ML) index has a problem that makes it hard to solve linear programming problems. To fix this, we created the GML index in this study using the directional distance function SBM, which is also known as GTFP. Here are the model’s specifications:


$$GTFP_{t}^{t+1}=\frac{1+\vec{S_{V}^{G}}\left(x^{t},y^{t},b^{t};g^{x},g^{y},g^{b}\right)}{1+\vec{S_{V}^{G}}\left(x^{t+1},y^{t+1},b^{t+1};g^{x},g^{y},g^{b}\right)}$$ (3)

Where $\vec{S_{V}^{G}}\left(x^{t},y^{t},b^{t};g^{x},g^{y},g^{b}\right)$ indicates the SBM distance function, and GTFP stands for the relative change value in period t + 1 depending on period t.The GTFP index has a threshold value of 1, meaning that a value larger than 1 denotes an improvement in the province’s green productivity, a value less than 1 denotes a deterioration, and a value equal to 1 denotes a stable state of the province’s green productivity. The table below lists specific data sources and input-output variables.

### 3.2. Core independent variable: Higher education (HE)

Currently, there are three main categories of evaluation indicators used to assess the level of development of higher education in academia: first, the scale of higher education development, such as the number of graduates; second, the use of funding related to higher education development; and third, the development of comprehensive indicators to assess the level of higher education development. In order to accurately and thoroughly depict the comprehensive development level of regional higher education, this study chooses its indicators of higher education from a variety of aspects, taking into account the logical rationality and empirical reliability of the selection process. As a result, this study builds a regional higher education development indicator system (Table 2 for specific indicators) by starting from the perspective of the social functions of higher education in the three dimensions of talent cultivation, scientific research, and social services, in light of the idea that “education should be compatible with economic development.” Measurement method: All of the indicators in Table 2 are combined into a single indication as a stand-in variable for HE using the entropy value approach in the objective assignment method. This study also includes the time variable to enhance the entropy value approach and make the analysis results more realistic by making the assessment indicators of various years of higher education comparable. The entropy value method’s fundamental idea is that an indicator’s weight increases with the amount of useful information it offers [28]. The precise measurement methods are as follows:

**Table 2:** Higher education evaluation indicator system.

Primary Indicators	Secondary indicators	Tertiary indicators	Indicator properties
Higher education	Talent cultivation	General undergraduate enrollment	+
		Number of full-time faculty members at the headquarters of higher education institutions	+
		Per capita funding for general colleges and universities	+
	Scientific research	Funding for R&D projects in higher education	+
		Number of monographs on scientific and technological achievements in higher education	+
		Number of papers on scientific and technological achievements in higher education	+
	Social Contributions	Participation rate of employed persons in continuing education	+
		Actual income from university technology transfer for the year	+
		Number of projects on the application of R&D results and scientific and technological services in higher education institutions	+

1. Standardization of indicators: This study used the approach of extreme variance for all indicators in order to remove the influence of scale and order of magnitude. Positive indicators among them are:

$$X_{it,j}^{'}=\frac{X_{it,j}-\min\left\{X_{it,j}\right\}}{max\left\{X_{it,j}\right\}-\min\left\{X_{it,j}\right\}}$$ (4)

Negative indicators are:


$$X_{it,j}^{'}=\frac{max\left\{X_{it,j}\right\}-X_{it,j}}{max\left\{X_{it,j}\right\}-\min\left\{X_{it,j}\right\}}$$ (5)

2. Determine the weights. These are the weights assigned to the jth indicator value, which represents the ith province’s level of higher education progress in the tth year.

$$P_{it,j}=\frac{X_{it,j}^{'}}{\sum_{i=1}^{N}\sum_{t=1}^{T}X_{it,j}^{'}}$$ (6)

3. Determine the redundancy and information entropy. The information entropy and redundancy of the jth indicator of the ith province’s higher education development level in the tth year is shown below.

$$e_{j}=-\frac{1}{\ln\left(NT\right)}\overset{N}{\underset{i=1}{\sum }}\overset{T}{\underset{t=1}{\sum }}P_{it,j}\ln\left(P_{it,j}\right)$$ (7)

$$d_{j}=1-e_{j}$$ (8)

4. Using the information entropy’s redundancy, get the weight of the jth indicator:

$$\omega_{j}=\frac{d_{j}}{\sum_{j=1}^{m}d_{j}}$$ (9)

5. Determine the level of development of higher education.

$$HE_{it}=\overset{m}{\underset{j=1}{\sum }}\omega_{j}X_{it,j}^{'}$$ (10)

### 3.3. Econometric models

The basic econometric model is shown below:


$$GTFP_{it}=\beta_{0}+\beta_{1}HE_{it}+\beta_{2}X_{it}+f\left(t\right)+\alpha_{i}+\varepsilon_{it}$$ (11)

where $GTFP_{it}$represents province I’s GTFP for year t. Higher education is indicated by${\text{HE}}_{it}$. One control variable is$X_{it}$ The temporal effect is represented by the third-order polynomial function f(t) [41,42]. The error term is$\varepsilon_{it}$ while the individual fixed effect is$\alpha_{i}$. The following variables are used as control variables: digital economy (Digital), industrial structure upgrading (Industry), international trade dependency (Trade), green finance (G-Finance), innovation output (Patent), and regional economic development level (PGDP). Regarding data sources and control variable measures, see section 4.2.

According to the mechanism analysis in hypothesis H_2_, higher education, industrial structure upgrading through technological and resource allocation advantages, and so on can all contribute to enhanced green productivity. In order to significantly contribute to increased green productivity, higher education and industrial structural upgrading must maintain a positive interaction within a specific range. Furthermore, higher education’s promotion of green production benefits from the positive moderating impact of the digital economy. The growth of the digital economy reduces barriers to technology and communication, improves resource accessibility, speeds up the flow of innovation factors like technology, information, talent, and capital, makes it easier to bridge the digital divide, and multiplies the impact of data on other innovation factors [43], all of which support the study and advancement of green technologies [44]. Furthermore, tertiary education ensures green innovation in the digital environment and supplies a skill pool to satisfy the demands of the digital economy’s expansion. Based on hypotheses H_2a_ and H_2b_, we configure model Eq. 12 to investigate the impact of the digital economy and the upgrading of industrial structures on green productivity in higher education.


$$GTFP_{it}=\beta_{1}HE_{it}+\beta_{2}\left(HE_{it}\times Z_{it}\right)+\gamma^{\prime }X_{it}+f\left(t\right)+\alpha_{i}+\varepsilon_{it}\left(12\right)$$ (12)

Where $Z_{it}$ in Eq. 12 denotes Digital and Industry, respectively, while the remaining variables are the same as in Eq. 11.

Based onhe mechanistic analysis in Hypothesis H_3_ and Eq. 11, the test taker can investigate the relationship between HE and GTFP in greater detail, taking into account the heterogeneous effect of higher education according to geographic income levels. Scholars like [45] have long recognized the importance of human capital endowment in influencing the regional GDP gap. They contend that there is a relationship between a region’s level of economic growth and the returns associated with its educational attainment. As a result, the research formulated the equation as follows to investigate the process underlying the role of income:


$$GTFP_{it}=\beta_{1}HE_{it}+\beta_{2}\left(HE_{it}\times D_{it}\right)+\gamma^{\prime }X_{it}+f\left(t\right)+\alpha_{i}+\varepsilon_{it}$$ (13)

where $D_{it}$ is a dummy variable that, in the event that the PGDP is higher than the median, assumes the value of 1 [42]. The interaction term between $HE_{it}$ and $D_{it}$ is $HE_{it}\times D_{it}$ A positive $\beta_{2}$ indicates that HE has a more linear effect on provinces with incomes above the median. The remaining variables in Eq. 11 are the same. The test taker then employs the interaction term directly between HE and income level, starting with the absolute income level, as the use of dummy variable estimation runs the risk of skewing the results:


$$GTFP_{it}=\beta_{1}HE_{it}+\beta_{2}\left(HE_{it}\times lnPGDP_{it}\right)+\gamma^{\prime }X_{it}+f\left(t\right)+\alpha_{i}+\varepsilon_{it}$$ (14)

Alternatively, we consider relative income levels:


$$GTFP_{it}=\beta_{1}HE_{it}+\beta_{2}\left(HE_{it}\times incgap_{it}\right)+\gamma^{\prime }X_{it}+f\left(t\right)+\alpha_{i}+\varepsilon_{it}$$ (15)

The ratio of GDP per capita to its greatest value, or $incgap_{it}=PGDP_{it}/\text{Max}\left(PGDP_{it}\right)$ is used to define the income gap [42]. The impact of HE is a linear function of income level in Eqs. 14 and 15. Positive $\beta_{2}$ indicates that provinces with higher income levels are more affected by HE.

However, there could be misspecification issues with the models in Eqs. 13–15. While the influence of HE on GTFP is based on a linear construction, it may not always follow a linear pattern. For instance, the impact coefficients varied by province, with marginal increments that vary with income level. In comparison, the partial linear function coefficient model (PLFC) is more adaptable, encompassing both a linear and nonlinear structure while accounting for variation across provinces and time [42,46]. The results and discussion in Section 5 measure specific provincial differences as well as time-varying disparities. This paper’s data structure and features have led to the specific setting of the PLFC model with fixed effects as follows:


$$GTFP_{it}=g\left(lnPGDP_{it}\right)HE_{it}+\gamma^{\prime }X_{it}+f\left(t\right)+\alpha_{i}+\varepsilon_{it}$$ (16)

Reference to [42], the nonparametric element of Eq. 16 is called $g\left(lnPGDP_{it}\right)$ and it represents the nonparametric effect of HE, which is an unknown function of the variable $lnPGDP_{it}$ In addition, $\gamma^{\prime }X_{it}$ represents the linear component of the required influences governing $GTFP_{it}$ Furthermore, the definition of every variable is the same as it is in Eq. 11.

## 4. Descriptive statistics and data sources

### 4.1. Dependent variable data sources

The research used panel data for 30 Chinese provinces from 2002 to 2021 (excluding Hong Kong, Macao, Taiwan, and Tibet due to data availability) to measure green productivity (GTFP). The study sourced all data from the China Statistical Yearbook, China Environmental Statistical Yearbook, and China Energy Statistical Yearbook, supplementing any missing information with provincial statistical yearbooks and bulletins on national economic and social development.

### 4.2. Data sources for core independent and control variables

The research used panel data covering 30 Chinese provinces from 2003 to 2021, excluding China, Hong Kong, Macao, Taiwan, and Tibet due to data availability, to measure the key independent variable, higher education, and the control variables. The National Statistical Bulletin on the Implementation of Education Expenditures, the Compendium of Higher Education Institutions Science and Technology Statistics, and the China Education Statistical Yearbook are the sources of the data for the three main independent variables, respectively.

The control variables PGDP, Digital, Trade, G-Finance, Industry, and Patent can be found in the China Statistical Yearbook, China Energy Statistical Yearbook, China Environmental Statistical Yearbook, China Internet Development Statistical Report, and statistical bulletins from the National Bureau of Statistics (NBS), provincial bureaus of statistics, and local governments. We use the interpolation method to fill in the few missing pieces of data. The specifics are described below:

1. Economic development level (PGDP). In contrast, regions with higher levels of economic development will have more funds to invest in green technology, which is more conducive to the development of the green economy. This is because Chinese cities have a significant GDP disparity, forcing regions with lower levels of economic development to rely on secondary industries to drive economic growth. Consequently, it is necessary to take into account the variability that various regions may display.
2. Dependency on foreign trade (Trade). We measure it by looking at the GDP to total import and export trade ratio. This statistic shows how open a region is to the outside world; the more often a region exchanges capital and technology with the rest of the globe, the more advanced its economy, innovation, and technology are.
3. Green Finance (G-Finance). Green finance has the power to redirect resources away from high-energy and pollution-consuming sectors and into more energy-efficient and environmentally friendly sectors. It can also force businesses to prioritize environmental protection, educate consumers about green consumption practices, and eventually lead to green development realization. Green credit, green investment, green insurance, and government support are the four indicators that make up the green finance index that is measured using the entropy technique [47].
4. Industrial structure upgrading (Industry). The development of the tertiary industry has a significant impact on clean and green production [48]; thus, this article, which refers to [49], uses the ratio of the tertiary industry’s output value to the secondary industry’s output value as a measure.
5. Innovation output (Patent). According to previous research, there is currently no single standard for measuring this indicator. For this reason, this paper uses the number of patents as a proxy for the innovation output indicator because it is deemed to be more representative and persuasive [50]. However, if this metric is overly pursued, it may result in the inclusion of low-quality and low-technology innovation outputs in the statistics, potentially harming the productivity of the green total factor.
6. Innovation output (Patent). According to previous research, there is currently no single standard for measuring this indicator. Because it is considered more representative and persuasive, this paper uses the number of patents as a proxy for the innovation output indicator [50]. However, excessive pursuit of this metric could lead to the inclusion of low-quality and low-technology innovation outputs in the statistics, potentially harming the productivity of the green total factor.

### 4.3. Descriptive statistics

Table 3 displays the descriptive statistics for the major variables. The GTFP ranges from 1.840 to 0.779, with a mean value of 1.143 and maximum and minimum values, respectively. Higher GTFP levels indicate higher productivity. However, there is also a correlation between higher wanted output and higher non-desired output. HE has a maximum value of 0.990 and a lowest value of 0.405. Therefore, it is evident that there exist regional disparities in China as a result of uneven regional growth based on the significant discrepancies between the maximum and minimum values of GTFP and HE. Furthermore, after conducting an empirical analysis in the upcoming section, the test taker discovered that the control variables have logarithmic values.

**Table 3:** Descriptive statistics of variables.

Variable	Mean	P50	Max	Min	sd	N
GTFP	1.143	1.121	1.840	0.779	0.125	570
HE	0.626	0.610	0.990	0.405	0.099	570
Digital	0.228	0.214	0.406	0.132	0.051	570
Trade	30.504	17.332	154.787	4.057	30.009	570
Industry	1.840	1.697	5.002	1.230	0.542	570
G_Finance	0.377	0.354	1.043	0.241	0.102	570
Patent	390.086	123.582	7040.225	0.775	716.140	570
PGDP	430.407	380.645	1839.800	36.860	301.833	570
GTFP2	1.080	1.080	1.822	0.733	0.109	570

## 5. Results and discussion

### 5.1. Benchmark model regression results

Table 4 displays the regression results for the benchmark model. Our study’s main concern is how higher education affects green productivity. Column (1) includes only the progress of higher education, while columns (2) through (6) include the control variables, the Digital Trade Industry G-Finance Patent.

**Table 4:** Estimation results of baseline model (dependent variable: GTFP).

Variable	(1)	(2)	(3)	(4)	(5)	(6)
HE	0.447***	0.461***	0.444***	0.436***	0.416***	0.433***
	(0.054)	(0.048)	(0.046)	(0.047)	(0.048)	(0.050)
Digital		0.379***	0.371***	0.377***	0.360***	0.341***
		(0.072)	(0.071)	(0.073)	(0.073)	(0.072)
Trade			0.083**	0.084**	0.067**	0.063*
			(0.036)	(0.035)	(0.033)	(0.034)
Industry				0.044**	0.038**	0.039**
				(0.018)	(0.018)	(0.017)
G_Finance					0.107***	0.106***
					(0.032)	(0.032)
Patent						0.033*
						(0.017)
Cons	0.765***	1.314***	1.086***	1.076***	1.224***	0.949***
	(0.028)	(0.113)	(0.160)	(0.161)	(0.160)	(0.207)
f(t)	Yes	Yes	Yes	Yes	Yes	Yes
Observations	570	570	570	570	570	570
Number of id	30	30	30	30	30	30

Note: Standard errors in parentheses.

*p <  0.1,

**p <  0.05,

***p <  0.01

HE coefficients in table (Table 4)'s columns are all significant and positive, suggesting that higher education encourages the rise of green productivity. This supports the new economic growth theory’s assertion that higher education plays a significant role in economic development and growth promotion [51]. Higher education helps to attain the Sustainable Development Goals (SDGs) [52,53], and it is a crucial driver of green economic growth, according to academicians [54]. Higher education reduces emissions by promoting green technology to combat pollution. On the one hand, it reduces energy consumption and increases energy efficiency through creative technological advancements.

The digital economy has a large and positive impact on green productivity for the control variables in columns (2) to (6). This result is consistent with findings from related studies conducted by academics like [11]. In addition to a digital platform that can fully dock market demand and supply information, the promotion and application of digital technology will enable businesses to optimize the efficiency of their own resource allocation, either actively or passively. This will significantly lower operating costs for businesses and improve resource allocation for society as a whole. All things considered, this will greatly reduce the depletion of natural resources and contamination of the ecological environment due to economic growth. Furthermore, the deep integration of the digital and real economies can effectively drive the intelligent digital transformation of traditional industries [43]. This integration can foster low-carbon, innovative, and sustainable production while optimizing resource utilization. The impact of international trade on green productivity is positive and large, as seen in columns (3) to (6). This is consistent with the findings of scholars [55] that foreign trade can significantly contribute to green development. In addition to being an effective means of achieving China’s carbon peak and carbon neutrality goals, promoting the green development of international trade is also a vital component of addressing global “green barriers.” To support the green development of foreign trade in the new development stage, it is recommended that trade adhere to the innovation drive, apply the new development concept, and foster the coordinated development of economic construction and ecological civilization at a deeper and higher degree of openness. The impact of green finance on green production development is also noteworthy and favorable, as seen in columns (5) to (6). This is consistent with the views of academicians [56], who believe that green finance promotes green transformation and economic development upgrading. The achievement of carbon peak and carbon neutrality targets is heavily reliant on green finance [57], which calls on banks to actively assist businesses in restructuring their energy-efficient and emission-reduction production lines, promote low-carbon and green initiatives, and participate in carbon emissions trading.

Furthermore, because of China’s enormous size and population, its regions differ greatly from one another in terms of their natural environments and development bases. Therefore, human beings must carefully consider addressing the uneven, inadequate, and disorganized development of China’s regions to achieve high-quality economic development. Therefore, in Section 5.3, the test taker scrutinizes the impact of income level on the relationship between HE and GTFP, considering the close connection between the unlocking of HE’s intrinsic potential and the foundation of regional economic development.

### 5.2. Model results for HE with ${\text{D}}_{\text{it}}$ , Industry, and Digital interaction terms

Table 5 displays the model’s estimation findings, which include the HE interaction variables with D_it_, Industry, and Digital. Columns (7) and (9) relate to Eqs. 13 and 12 to column (7). The dummy variable (D_it_) and HE interact in column (7), and the interaction term’s coefficient is considerably positive, suggesting that the greater the relevance of HE’s effect on GTFP in provinces with higher income levels, Column (8) contains the interaction term between HE and Industry. Its coefficient is significantly positive, suggesting that industry upgrading positively influences the link between HE and GTFP. Column (9) contains the interaction term between HE and Digital, displaying a significantly positive coefficient. This suggests that the digital economy has a more pronouncedly empowering effect on green productivity, as well as a strong and significant positive effect on the relationship between HE and GTFP.

**Table 5:** Results of models with interactive terms (dependent variable: GTFP).

Variable	(7)	(8)	(9)
HE	0.294***	0.394***	1.267***
	(0.043)	(0.059)	(0.156)
HE × D	0.205***		
	(0.016)		
HE × Industry		0.056*	
		(0.028)	
HE × Digital			0.548***
			(0.102)
Digital	0.265***	0.342***	
	(0.061)	(0.072)	
Trade	0.079***	0.064*	0.065*
	(0.025)	(0.034)	(0.034)
Industry	0.018		0.040**
	(0.015)		(0.016)
G_Finance	0.092***	0.106***	0.108***
	(0.031)	(0.032)	(0.031)
Patent	0.032**	0.033*	0.035**
	(0.015)	(0.017)	(0.017)
Cons	0.854***	0.971***	0.412**
	(0.137)	(0.208)	(0.152)
f(t)	Yes	Yes	Yes
Observations	570	570	570
Number of id	30	30	30

Note: Standard errors in parentheses.

*p <  0.1,

**p <  0.05,

***p <  0.01

### 5.3. Model results for the HE and income interaction term

Table 6 displays the model’s estimation findings, which include the interaction term between HE and income. Eqs. 14 and 15 correspond to columns (10) and (11). The estimation results of the linear portion of the partial linear function coefficients model in Eq. 16, which are covered in Section 5.4, are correlated with Column (12).

**Table 6:** Results of models with interactive terms between HE and income (dependent variable: GTFP_1_).

Variable	(10)	(11)	(12)
HE	−0.193	0.430***	
	(0.311)	(0.063)	
HE × lnPGDP	0.102**		
	(0.049)		
HE × incgap		0.013	
		(0.093)	
Digital	0.325***	0.341***	0.326***
	(0.068)	(0.072)	(0.094)
Trade	0.063*	0.062*	0.484***
	(0.036)	(0.036)	(0.109)
Industry	0.038**	0.039**	0.012
	(0.016)	(0.017)	(0.021)
G_Finance	0.106***	0.107***	0.085***
	(0.031)	(0.032)	(0.031)
Patent	0.026	0.033*	0.123***
	(0.018)	(0.017)	(0.033)
Cons	1.071***	0.951***	
	(0.241)	(0.215)	
f(t)	Yes	Yes	Yes
Observations	570	570	540
Number of id	30	30	30

Note: Standard errors in parentheses.

*p <  0.1,

**p <  0.05,

***p <  0.01

In concentrate on the interaction term between HE and the income proxy factors in the empirical data shown in Table 6. Column (10) includes the relationship between HE and GDP per capita, and the interaction term’s coefficient is considerably positive, suggesting that the influence of HE on GTFP is stronger in provinces with greater absolute income levels. Additionally, column (11) adds the interaction term between HE and absolute income level, but the coefficient remains non-significant.

The estimates in Table 6’s columns (10) and (11) show that alternative model setups yield wildly divergent estimation findings. This discrepancy suggests that the conventional linear function model might not be able to accurately depict the exact mechanism underlying HE’s impact on GTFP. Based on this, in Section 5.4, the research will estimate HE’s course of action on GTFP using the PLFC model with spatio-temporal heterogeneity and linear and non-linear properties.

### 5.4. Estimation results of the PLFC model

**Fig 1:**
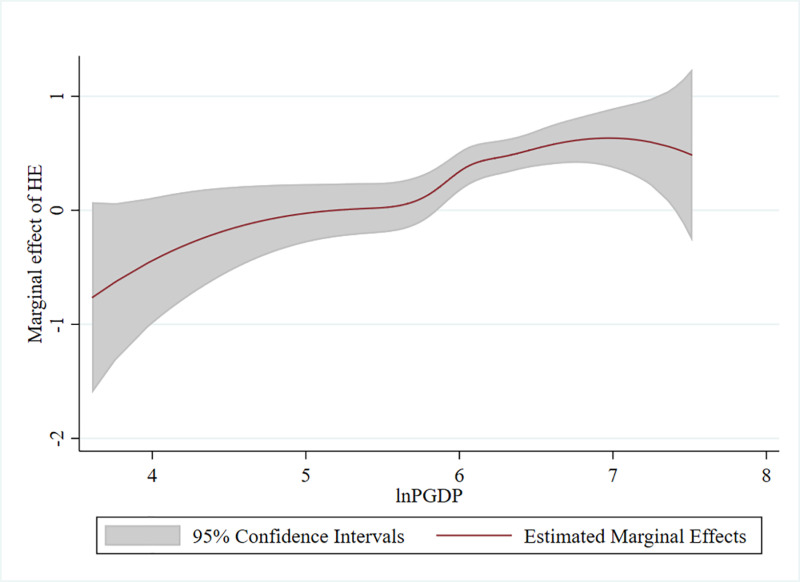
Estimated function coefficients. Note: Lines indicate estimated function coefficients and blue-gray shaded areas indicate 95% confidence intervals.

The research utilizes the partial linear function coefficients panel model of Eq. 16 to prevent modeling mistakes, and Fig 1 provides the estimation results of the nonparametric component of HE, or $g\left(lnPGDP_{it}\right)$ The estimation results of the linear component are provided in Table 5’s column (12), and as can be seen, they are largely consistent with the traditional model’s results in terms of significance and conformance. The empirical results for the nonparametric component, which differs from the conventional linear model in terms of information, are displayed in Fig 1.

To avoid modeling mistakes, we use the partial linear function coefficients panel model of Eq. 16, and Fig 1 provides the estimation results of the nonparametric component of HE, or $g\left(lnPGDP_{it}\right)$ Table 5’s column (12) provides the estimation results of the linear component, which are largely consistent with the results of the traditional model in terms of significance and conformance. Fig 1 displays the empirical results for the nonparametric component, which differs from the conventional linear model in terms of information.

Next, the research investigates the PLFC model’s findings using three different methods. First, GDP per capita determines how significant HE impact is. Fig 1 splits the magnitude of HE’s impact into two categories. In particular, when the logarithm of GDP per capita (lnPGDP) is less than 5.823, the predicted function coefficients’ 95% confidence interval is zero, indicating that HE has a negligible influence. Conversely, the projected effect of HE is significant at the 5% level of significance when the value of lnPGDP is higher than 5.823. This means that only when GDP per capita exceeds a certain threshold can the marginal impact of HE on green production become substantial.

Second, as GDP per capita increases, so does the marginal green productivity growth effect of HE. The marginal effect of HE is represented by the red line in Fig 1, which shows an increasing tendency with GDP per capita increase in the early period but a declining trend after 2019 because of the global New Crown Pneumonia outbreak.

Third, the empirical findings of the PLFC model demonstrate that the importance and strength of HE’s effect differ across provinces. On the other hand, the interaction term between HE and income has no quantifiable impact using the conventional linear model in Table 3. In particular, when GDP per capita surpasses a threshold number, the marginal effect of HE on green productivity rises with the level of GDP per capita.

This is consistent with [58]’s view that the role of higher education (HE) on green development is influenced by the level of economic development.

**Fig 2:**
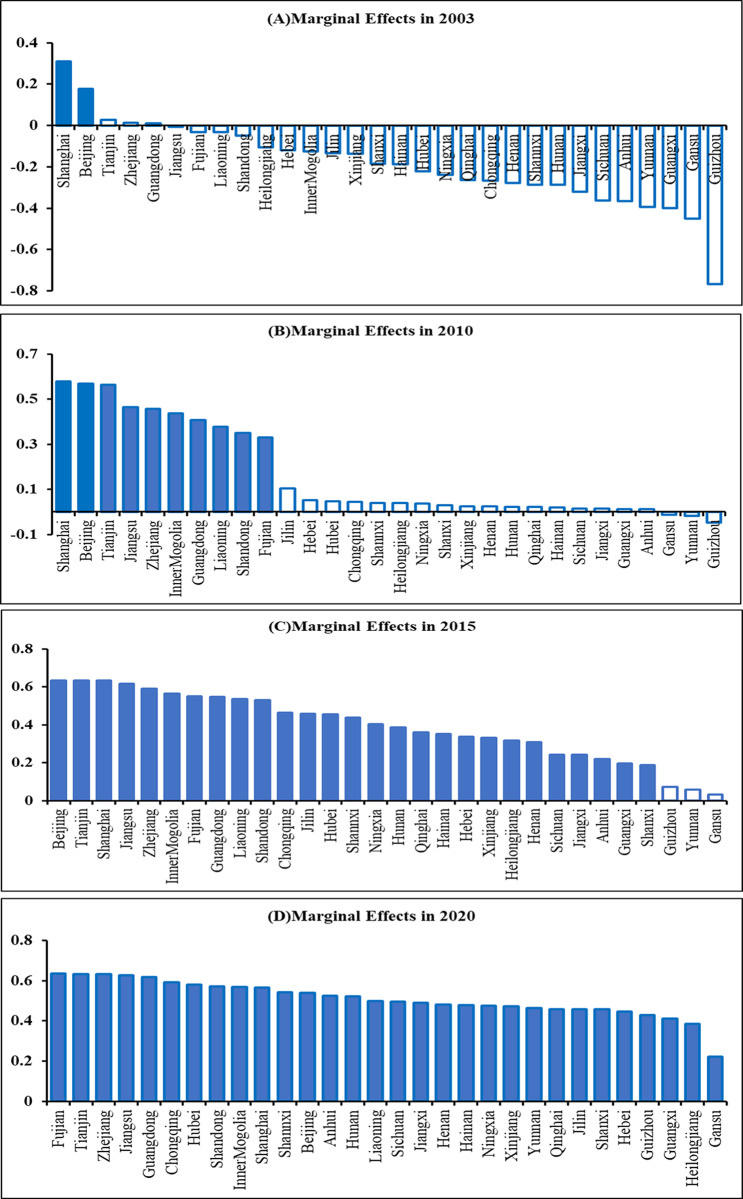
Marginal effects by province over the years. Note: Solid and hollow bars indicate significant and non-significant effects at the 5% level, respectively.

Using [42] as a guide, the test taker then calculates the marginal effect of HE on GTFP growth over time and show the results for normal years in Fig 2. This is because Chinese provinces’ natural conditions and development foundations are very different. The number of provinces with marginal and significant HE impacts has been rising over time, according to the overall A-D results in Fig 2. In 2003, the majority of provinces experienced negative effects, with only two having large and positive marginal effects. But as time goes on, by 2020, the provinces’ HE’s marginal effect will be substantial and positive, and the difference between the total level of provinces and the impact of green production will be closing. The plot of marginal effects by province in 2021, which is not displayed here, demonstrates that the marginal effects of the cosmopolitan cities of Beijing and Shanghai are insignificant, which is most likely due to the global new crown epidemic.

### 5.5. Robustness tests

Based on the empirical research above, individual knows that HE can significantly increase GTFP, and provinces with higher GDP per capita can amplify HE’s promotion effect on GTFP. Next, the test taker uses a threshold model in place of the dependent variable to test the model’s robustness.

The research uses the SBM-DEA model to recalculate GTFP as an explanatory variable in the regressions of the benchmark and PLFC models because the way it is measured can change. Table 7 presents our empirical results. According to [59], the regression results of the threshold model are displayed in columns (15) and (16). Column (13) corresponds to the empirical results of the linear part of the PLFC model in Eq. 11 of the benchmark model, while column (14) corresponds to the empirical results of the linear part of the PLFC model in Eq. 16.

**Table 7:** Results of the robustness test.

Dependent variable	GTFP_1_		GTFP (Threshold variable: lnPGDP)	
	(13)	(14)	(15)	(16)
HE	0.355***		HE(Th ≤ r)	0.264***
	(0.051)			(0.048)
Digital	0.348***	0.369***	HE(Th > r)	0.440***
	(0.073)	(0.108)		(0.045)
Trade	0.078**	0.540***	Digital	0.263***
	(0.032)	(0.121)		(0.046)
Industry	0.034*	0.017	Trade	0.054**
	(0.018)	(0.024)		(0.022)
G_Finance	0.114***	0.096***	Industry	0.002
	(0.036)	(0.035)		(0.016)
Patent	0.035*	0.137***	G_Finance	0.097***
	(0.017)	(0.038)		(0.018)
Cons	0.936***		Patent	-0.002
	(0.189)			(0.006)
f(t)	Yes	Yes	Threshold value	5.939
Observations	570	540	Observations	570
Number of id	30	30	Number of id	30

Note: Standard errors in parentheses.

*p <  0.1,

**p <  0.05,

***p <  0.01

**Fig 3:**
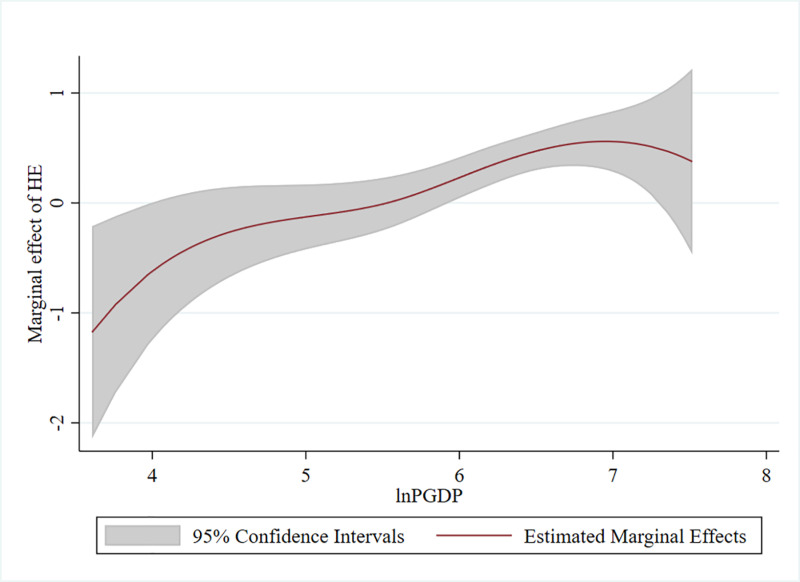
Estimates of the nonlinear part of the PLFC model in the robustness test. Note: Lines indicate estimated function coefficients and blue-gray shaded areas indicate 95% confidence intervals.

The results individual got from our experiments show that the linear part of the estimates in Table 7’s columns (13) and (14) is important, and its sign matches the earlier regression results. The computed $g\left(lnPGDP_{it}\right)$ coefficients, displayed in Fig 3, exhibit an increasing trend and sustain a considerable transition from negative to positive values. This indicates that as GDP per capita rises, the marginal incentive effect of higher education on GTFP initially decreases and then increases. The results in Fig 3, which corroborate our empirical results from Fig 1 in the preceding section, further validate the robustness of our results.

Using [60]᾿s method, the test taker constructs a fixed-effects panel threshold model for regression analysis to verify if distinct models are responsible for the discrepancies in the previous empirical results. In particular, we employ GDP per capita as the threshold variable. Table 7’s columns (15) and (16) display the results of our regression. When the threshold is higher, the marginal effect of higher education on GTFP is strongly positive and has a higher value (0.440), indicating that provinces with a higher GDP per capita make greater contributions to the development of green productivity. This is consistent with [58]’s findings that higher education in educationally developed regions has a more favorable impact on green development.

The research can say that the estimation results in this section pass the robustness test because they are similar to the results of the benchmark model and the PLFC model regression. This serves as more evidence of how crucial it is to support the coordinated growth of the local economy and educational system. Therefore, the institutional mechanism of continuous talent flow has a positive effect on the full development and improvement of the provinces’ economic efficiency, as well as the promotion of green development. Higher education, as one of the fundamental components of economic development, has a very important impact on the region’s coordinated development [61].

## 6. Conclusions and recommendations

### 6.1. Conclusion

Increasing the level of green total factor productivity is crucial for fostering green development, and as China’s reform and opening up continue, along with its emphasis on talent development, higher education development is becoming more and more important for the growth of green productivity. This study uses panel data from 30 Chinese provinces as its research subject, conducts an empirical test and theoretical analysis to determine how education affects regional green productivity, and draws the following conclusions:

Higher education raises the level of green productivity through industrial structure upgrading, at which time the direct effect exists simultaneously with the indirect effect, and although it contributes to the improvement of eco-efficiency, it has a weaker force. First, higher education, industrial structure upgrading, and the digital economy can all promote green productivity, respectively. After adding the two interaction terms, the above conclusion still holds, and the interaction term is significantly positive. The digital economy is different because it possesses a stronger force. Specifically, as it develops, it has the potential to raise the level of education development and increase regional green productivity. The application of digital technology fosters favorable conditions for the realization of inventive advancement, and the growth of the digital economy lowers all expenses associated with the innovation process. The digital economy has imposed higher standards for employee quality, leading to the transformation and upscaling of traditional industries and the emergence of new ones. This has the effect of encouraging workers to continually enhance their knowledge and abilities, which, in turn, has advanced higher education. When the two collaborate, the likelihood of enhancing green productivity increases.

Second, the PLFC model is used in this study because the link between higher education and regional green productivity is not linear and varies by region. The PLFC model can look at the link between higher education and income level as a non-linear function [62]. The study’s findings indicate that the marginal effect of higher education on green productivity growth increases with the level of regional economic development. Simultaneously, we computed the marginal impact of higher education development on the rise of green productivity in every region. The results in Fig 2 show that as time has gone on, both the number of provinces where higher education development has a significant impact and the marginal effect of higher education development have increased annually. These changes suggest that higher education development has a steadily growing influence on provincial green development.

### 6.2. Recommendations

Based on the aforementioned findings, this study makes the following recommendations for improving the use of higher education to increase green total factor productivity:

First, prioritize human capital development through education and talent deployment. For high-quality human capital to fully realize its worth and boost regional green productivity, China’s education system, particularly higher education, must be significantly reformed. Initially, in order to raise the educational bar for the labor force and swap out the quantitative dividend for the population’s qualitative dividend, education reform should increase the number of years of education per capita and deepen the development of a society that values lifelong learning. Furthermore, in order to cultivate high-level talent, the state should establish a scientific talent program. It should also always uphold the cultivation concept of “theory-practice” in the curriculum and teacher training, insisting on the close integration of vocational education and work practice, exploring and deepening theories, and encouraging the coordinated development of higher education. In conclusion, it is imperative that we construct and enhance the regional talent market mechanism, offer appropriate development spaces and platforms for talent at varying levels, optimize the regional talent aggregation structure, and elevate the overall quality of China’s human capital in higher education.

Second, human beings should accelerate the digital economy’s development by implementing the new “digital + talent” development model. The green innovation impact of the digital economy is intrinsically linked to the investment in high-quality human capital. For underdeveloped regions with educational deficiencies, the government must persist in enhancing higher education and vocational training to address the talent deficit in the digital economy and green innovation, thereby maximizing its potential for boosting green innovation [43]. For the developed regions, it is crucial to continue enhancing the talent cultivation system and investing in human capital education with a high-level talent focus. One cannot fully separate the impact of the digital economy on green innovation from the support of human capital investment. To solve the “necklace” problem of the development of digital technology and support the growth of the green economy, developed regions must continue to improve the talent training system, direct human capital investment in education towards high-level talents, particularly top-notch talents and scientific and technological leaders, and effectively improve the overall level of higher education development.

Third, encourage the modernization of the industrial structure and improve the relationship between it and the advancement of higher education [51]. Despite their synergy’s potential to boost green productivity, its impact has been minimal so far. However, modernizing industrial structures can moderate the impact of higher education on green productivity. Therefore, it is crucial to take into account the objectives of the local industrial structure and talent pool when making adjustments to higher education development. If the skills required to modernize the industrial structure are insufficient to sustain development, it is imperative to conduct prospective training ahead of time for the categories of human capital where a sizable deficit exists. For instance, if there is a clear shortage of skilled labor in higher education, people should encourage schools to better target human capital training. They should also concentrate on the overcultivation or under cultivation of a particular type of human capital and work to find an equilibrium point for human capital training. Therefore, to optimize the effectiveness of green development, it is crucial to regularly adjust the dynamic adaptation of higher education development and industrial structure upgrading during the economic development process.

This paper has some limitations. First, the focus of this study is on China; however, in order to contribute to broader theories and debates in the field of higher education and green production, this study could be expanded to other nations, including Asian and/or global perspectives. Second, given the spatial spillover effects of higher education development, spatial regression modeling could be used to investigate the mechanisms behind their interaction.

## Supporting information

S1 File Data set. (XLSX)
